# Active induction of experimental autoimmune encephalomyelitis by MOG_35-55_ peptide immunization is associated with differential responses in separate compartments of the choroid plexus

**DOI:** 10.1186/2045-8118-9-15

**Published:** 2012-08-07

**Authors:** Nivetha Murugesan, Debayon Paul, Yen Lemire, Bandana Shrestha, Shujun Ge, Joel S Pachter

**Affiliations:** 1 Blood–brain Barrier Laboratory, Department of Cell Biology, University of Connecticut Health Center, 263 Farmington Ave, Farmington, CT, 06030, USA

**Keywords:** Laser capture microdissection (LCM), Choroid plexus, EAE, Pertussis toxin, Neuroinflammation

## Abstract

**Background:**

There is increasing awareness that, aside from producing cerebrospinal fluid, the choroid plexus (CP) might be a key regulator of immune activity in the central nervous system (CNS) during neuroinflammation. Specifically, the CP has recently been posited to control entry of sentinel T cells into the uninflamed CNS during the early stages of neuroinflammatory diseases, like multiple sclerosis (MS) and its animal model experimental autoimmune encephalomyelitis (EAE). As the CP is compartmentalized into a stromal core containing fenestrated capillaries devoid of typical blood–brain barrier properties, surrounded by a tight junction-expressing choroidal epithelium, each of these compartments might mount unique responses that instigate the neuroinflammatory process.

**Methods:**

To discern responses of the respective CP stromal capillary and choroidal epithelial tissues during evolving neuroinflammation, we investigated morphology and *in situ* expression of 93 immune-related genes during early stages of EAE induced by immunization with myelin oligodendrocyte glycoprotein peptide (MOG_35-55_). Specifically, 3-D immunofluorescent imaging was employed to gauge morphological changes, and laser capture microdissection was coupled to an *Immune Panel* TaqMan Low Density Array to detail alterations in gene expression patterns at these separate CP sites on days 9 and 15 post-immunization (p.i.). To resolve CP effects due to autoimmunity against MOG peptide, from those due to complete Freund’s adjuvant (CFA) and pertussis toxin (PTX) included in the immunization, analysis was performed on MOG-CFA/PTX-treated, CFA/PTX-treated, and naïve cohorts.

**Results:**

The CP became swollen and displayed significant molecular changes in response to MOG-CFA/PTX immunization. Both stromal capillary and choroidal epithelial tissues mounted vigorous, yet different, changes in expression of numerous genes over the time course analyzed - including those encoding adhesion molecules, cytokines, chemokines, statins, interleukins, T cell activation markers, costimulatory molecules, cyclooxygenase, pro-inflammatory transcription factors and pro-apoptotic markers. Moreover, CFA/PTX-treatment, alone, resulted in extensive, though less robust, alterations in both CP compartments.

**Conclusions:**

MOG-CFA/PTX immunization significantly affects CP morphology and stimulates distinct expression patterns of immune-related genes in CP stromal capillary and epithelial tissues during evolving EAE. CFA/PTX treatment, alone, causes widespread gene alterations that could prime the CP to unlock the CNS to T cell infiltration during neuroinflammatory disease.

## Introduction

Though the choroid plexus (CP) is commonly recognized as the production site of cerebrospinal fluid (CSF) [1-3], it has relatively recently gained attention as a critical player in central nervous system (CNS) inflammation [4-6]. Specifically, the CP has been suggested as the site of entry into the uninflamed CNS of pioneer T cells searching for their cognate antigens during immunosurveillance and in the early stages of neuroinflammatory diseases such as multiple sclerosis (MS) and its animal model experimental autoimmune encephalomyelitis (EAE) [7,8]. Current theory holds that, after crossing the CP into the CSF, pioneer T cells travel to the subarachnoid space (SAS), where antigen-presenting cells reactivate them. In turn, reactivation is thought to set off a burst of cytokines and other mediators that inflames meningeal and parenchymal venules to initiate disease [9-11].

The anatomy of the CP appears well suited to orchestrating the initial steps of CNS inflammation. It projects from the roofs of all four ventricles into the CSF, and is composed of two distinct tissue layers: a highly vascularized stroma encapsulated by a “tight” layer of epithelial cells [12]. Unlike the parenchymal capillaries forming the restrictive blood–brain barrier (BBB), CP stromal capillaries are fenestrated and contain pentilaminar junctions whose outer leaflets are not fused [13] – properties that render the CP capillary population highly permeable to macromolecules [14]. This juxtaposition of “leaky” capillaries and tight epithelium constitutes the blood-cerebrospinal fluid barrier (BSCFB) [15,16], an arrangement construed as enabling blood-born leukocytes to extravasate into an uninflamed brain during the incipient stages of MS and EAE [17,18]. Supporting this process, expression of chemokine CCL20 by choroidal epithelial cells is thought to chemotactically draw T cells – bearing the cognate receptor CCR6 – from the CP stroma, across the epithelium and into the CSF [8].

*In situ* hybridization and immuno-electron microscopy of the CP has further revealed expression of adhesion molecules, VCAM-1 and ICAM-1, by choroidal epithelial cells of the healthy CP, and additionally of MAdCAM-1 by these same cells during EAE [19,20]. Also, transcriptome analysis of the whole adult CP has highlighted expression of immune mediators in both healthy mice [21] and those subject to peripheral inflammation [22], reinforcing the view this organ is a critical conduit linking immune/inflammatory activities between the periphery and CNS. But the extremely close apposition of the different CP layers has posed a significant challenge to studying the depth of their respective contributions to inflammatory processes. In fact, no immune function has yet been ascribed to the CP capillary endothelium, leaving completely unresolved the factors that drive T cell emigration into the stroma. And gene regulatory events surrounding transmigration of T cells across the choroidal epithelium further remain unsettled.

To elaborate the sequence of events in the CP that set the stage for CNS inflammation during EAE induced by active immunization with MOG_35-55_ peptide, we used laser capture microdissection (LCM) coupled to qrt-PCR-based microarray [23] to establish the time course of expression of a panorama of immune mediators in the separate stromal (including capillaries) and choroid epithelial layers. Morphological changes in the CP associated with MOG immunization were also examined by quantitative 3-D image analysis following confocal microscopy. Results reveal substantial changes in CP gene expression and morphology occurred in response to vspecific aspects of the MOG immunization process. These results could hold relevance for how combinations of environmental factors trigger neuroinflammatory disease.

## Materials and methods

### Animals

Female C57BL/6 mice, age 8–10 weeks and obtained from Charles River Laboratories, Inc. (Wilmington, MA), were used to minimize microvascular heterogeneity due to genetic variability, sex, and age [24]. Animals were euthanized by CO_2_ inhalation, following Animal Care and Use Guidelines of the University of Connecticut Health Center (Animal Welfare Assurance # A3471-01). A total of n = 3 animals/group were used for each treatment and time-point assessed.

### Induction of experimental autoimmune encephalomyelitis (EAE)

EAE was induced in mice by active immunization with MOG_35-55_ peptide (MEVGWYRSPFSRVVHLYRNGK), of murine origin (W. M. Keck Biotechnology Resource Center, Yale University), as described [25]; following Animal Care and Use Guidelines of the University of Connecticut Health Center (Animal Welfare Assurance # A3471-01). Briefly, on day 0, one group of female mice 7–9 weeks of age was injected subcutaneously with 300 μg of MOG peptide in complete Freund’s adjuvant (CFA, DIFCO) into the right and left flank, 100 μl per site. These mice were also injected i.p. with 500 ng pertussis toxin (PTX, List Laboratories, Campbell CA) in PBS on days 0 and 2 following the first immunization (referred to as the MOG-CFA/PTX group). The second group of age-matched mice received CFA alone and PTX (500 ng) injections on day 0 and a second injection of 500 ng PTX alone on day 2 (referred to as the CFA/PTX group). The third group of naïve age-matched female mice was left untreated. Animals were monitored and scored daily for clinical disease severity according to the following scale: 0 = normal; 1 = tail limpness; 2 = limp tail and weakness of hind legs; 3 = limp tail and complete paralysis hind legs; 4 = limp tail, complete hind leg and partial front leg paralysis; and 5 = death. LCM tissue was acquired at day 9 (score 0) and day 15 (score ~ 2.0) post-immunizations.

### Tissue preparation for Immuno-LCM

Brains were snap-frozen in dry ice-cooled 2-methylbutane (Acros; Geel, Belgium), and stored at −80°C. Frozen brain was embedded in cryomatrix compound (Thermo Fisher Scientific, Waltham, MA) prior to sectioning. Coronal sections (7 μm) were cut on a Microm HM 505 M cryostat (Mikron Instruments; Oakland, NJ) and affixed to uncoated, pre-cleaned glass slides (Fisher Scientific, Pittsburgh, PA) and stored in a slide box at −80°C. Tissue was processed for LCM within a week of sectioning.

### Immunostaining for Immuno-LCM

Immunostaining was performed as detailed [24,26,27], with minor modifications. Briefly, sections were fixed in 75% ethanol, on ice, for 3 min prior to staining. The CP stromal capillaries were stained using alkaline phosphatase substrate NBT (nitro-blue tetrazolium chloride)/BCIP (5-bromo-4-chloro-3'-indolyphosphate p-toluidine salt), (Vector Labs, Burlingame, CA) for 3–5 minutes in 100 Mm Tris–HCl (pH 9.5) to detect endogenous alkaline phosphatase activity in the endothelial cells. In this case, endothelial cells were intentionally not immunostained by anti-CD31/ABC alkaline phosphatase [24,27], as it resulted in extensive deposition of chromogenic precipitate, which made the stromal capillaries difficult to resolve from the choroidal epithelial layer. The choroidal epithelial cells were immunostained with monoclonal pan-cytokeratin-FITC antibody (Sigma) for 10 minutes (diluted 1:10 in 1X PBS + 0.5% Tween-20). RNAsin® RNAse inhibitor (Promega, Madison, WI) was added to all staining reagents. Immediately after immunostaining, sections were dehydrated through graded alcohol and xylenes as described [24].

### Laser capture microdissection (LCM)

A PixCell IIe laser capture microscope (ABI, Foster City, CA) was used to separately procure CP stromal capillary and CP choroidal epithelial tissues, as previously described for brain parenchymal vessels [24,27,28]. We refer specifically to CP stromal capillary tissue, instead of pure capillary endothelium, as it was not possible to completely resolve vascular from matrix elements (including extravasating leukocytes) within the dense CP stroma. Likewise, the choroidal epithelial tissue may contain some epiplexus cells, and so is not described as pure epithelium. Only choroid plexus material from within the fourth ventricle and lateral recess of the fourth ventricle was retrieved.

### Tissue extraction

LCM-retrieved tissue was solubilized in Cell Lysate Buffer® (Signosis; Sunnyvale, CA) for direct reverse transcription. Cell Lysate Buffer®, pre-heated to 75°C, was added and the resulting lysate heated at 75°C for an additional 15 min. Samples were immediately frozen at −80°C.

### DNase treatment and cDNA synthesis

Cell Lysate Buffer® extracts were treated with Turbo DNase (Ambion; Austin, TX) according to the manufacturer’s instructions. Specifically, Turbo DNase buffer and DNase were added and samples incubated at 37°C for 30 min. Next, DNAse inactivation reagent was added for 2 min at room temperature. Samples were then reverse transcribed using the SuperScript III (Invitrogen) standard protocol with random hexamers (Roche; Indianapolis, IN), and employing an extension temperature of 42°C – optimal for random hexamers – for 60 min. Resulting cDNA was stored at −20°C until used for analysis.

### cDNA Pre-Amplification

Pre-amplification was carried for array analysis out using TaqMan® PreAmp Master Mix and a PreAmp Pool containing all the primers for detection by the Mouse Immune Panel TaqMan® Low density Array (TLDA; Life Technologies Corp., Foster City, CA) [23]. This panel conatins 93 immune-related genes plus three housekeeping control genes *(*see Additional file 1: *Mouse Immune Panel TLDA*). Pre-amplification was carried out with an initial hold at 95°C for 10 min, followed by 14 cycles at 95°C for 15 sec and 60°C for 4 min.

### qrt-PCR

Relative cDNA levels were quantified by qrt-PCR using an ABI PRISM 7500 Sequence Detection System Version 2.3, and reported compared to housekeeping gene GAPDH. Relative quantitation to GAPDH was performed using the standard 2^–δCt^ method of Pfaffl [29], where δCt = Ct target threshold cycle – Ct reference (GAPDH) threshold cycle. Expression of genes relative to GAPDH was then represented as percent expression of GAPDH. To assure consistency in relating gene expression patterns to a housekeeping gene, GAPDH and two other housekeeping genes, β-actin and 18 S ribosomal RNA, were evaluated for constant expression across treatments. Additionally, gene expression values for a handful of randomly selected immune-related genes were also determined relative to β-actin and 18 S ribosomal RNA (see Additional file 2: *Housekeeping control genes*)*.* Custom TaqMan® primers/probes were used for the Mouse Immune Panel TLDA. TLDA analysis was conducted as per the manufacturer’s protocol, with 100 μl sample volumes containing a 1/32 dilution of pre-amplified cDNA added to each port of the microfluidic card [23]. For *qrt-PCR* analysis of CD31 and Cytokeratin 8, ‘singleplex’ assays were used as neither of these genes are represented in the mouse Immune Panel TLDA.

### Immunostaining for confocal microscopy

Frozen cryosections (60 μm) were fixed with 4% paraformaldehyde, permeabilized with 1% Triton X-100 (in PBS) and incubated with Powerblock® for 10 min. Purified rat anti-mouse CD31 antibody (BD Pharmingen; 1:150 dilution in 10% NBS in 1X PBS + 0.5% TW-20) was used to stain the CP capillary network followed by incubation with goat anti-rat Alexa-555 secondary antibody (1:200). Pan-cytokeratin-FITC 1: 150 dilution, (Sigma) was used to stain the CP epithelium. Next, Alexa-647 anti-mouse CD45 antibody (1:160 dilution) was used to stain leukocytes.

### Confocal microscopy

Images were acquired on a Zeiss LSM 510 Meta laser scanning confocal microscope, and optical slices (at 2-μm intervals) obtained using a 40x objective. Acquired z-stacks were background-subtracted, and 3-D isosurface rendering performed using Bitplane IMARIS suite version 7.1 x 64 software (Bitplane Inc. Saint Paul, MN). Each z-stack was thresholded and the “filament tracker” module used to generate a 3-D traced outline of immunostained vessels in order to determine the diameter range of the CD31-immunostained capillary network within the choroid plexus across different treatments.

### Statistical analysis

Relative gene expression values are given as mean ± SEM. Student’s two-tailed test (Microsoft Excel 2003, Redmond, WA) was employed to assess statistical significance in gene expression values between MOG-CFA/PTX and CFA/PTX samples from the CP capillary stroma and CP epithelium groups, separately for the two different time points assessed. Results were considered significant at a *p* ≤ 0.05. Additionally, two-way ANOVA followed by post-hoc Bonferroni analysis was performed using GraphPad Prism 5 (GraphPad, La Jolla, CA) to determine *interactive effects* between immunization treatment and time of analysis post-immunization, and assessed for each CP compartment.

## Results

### Anatomy of the CP is altered in response to MOG-immunization

**Figure 1:**
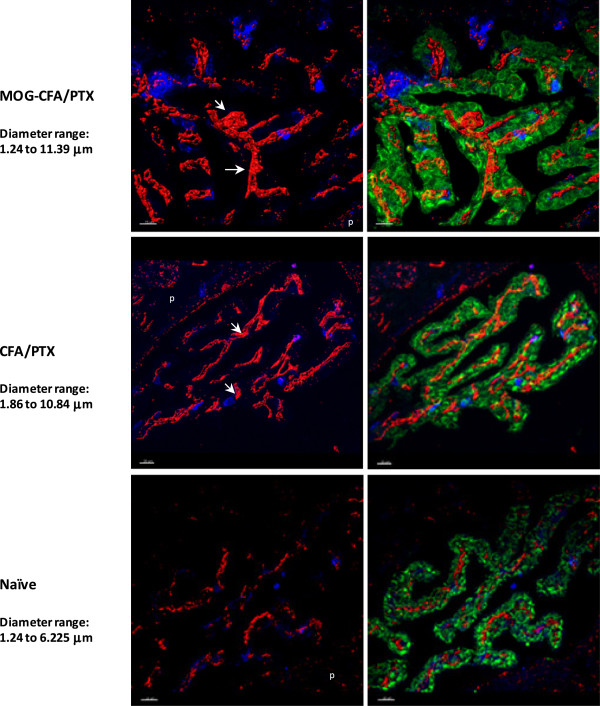
**Morphological analysis of CP compartments following immunization.** CP epithelium was stained with polyclonal antibody to pan-cytokeratin (FITC, green), and CP stromal capillaries were immunostained with monoclonal anti-CD31 antibody (red). A CD45 antibody was used to stain for any leukocytes present within the CP (blue). Confocal microscopy z-stack images of thick (60 μm) frozen sections of the CP were acquired, and three dimensional rendering was performed using Imaris image analysis software. Shown are rendered images of the CP from all three treatment conditions (MOG-CFA/PTX day 15 p.i., CFA-PTX day 15 p.i. and Naïve), revealing swelling of the stromal capillaries (arrows) following both immunization protocols. The left side shows leukocyte and capillary staining, emphasizing the distended capillary diameters. The right side is a composite of leukocyte, capillary, and epithelial staining. Mean capillary diameter ranges for each group were determined using the Filament tracer module in Imaris. The choroidal epithelium appears unaltered by immunization. ‘p’ indicates brain parenchymal region. Scale: 20 μm.

First, the anatomy of the CP was investigated using confocal microscopy followed by 3-D isosurface rendering. The close apposition of stromal capillary and choroidal epithelial layers in the CP is depicted in Figure 1. The 3-D analysis highlights the tortuosity of the capillary plexus. At day 15 post immunization (p.i.) with PTX and MOG_35-55_ peptide in CFA to induce EAE (MOG-CFA/PTX group), or with PTX and CFA alone (CFA/PTX group), which does not produce disease in this paradigm, the capillary plexus can be seen to locally ‘swell’ in certain regions (Figure 1). Specifically, the range in diameter of capillaries in the MOG-CFA/PTX and CFA/PTX groups was 1.24 to 11.39 μm and 1.86 to 10.84 μm respectively, as compared to that found in naïve (1.24 to 6.22 μm) mice. In contrast to that seen within CP capillaries, the morphology of the choroidal epithelial layer remained relatively constant following immunization.

### LCM enables resolution of CP stromal capillaries from the choroidal epithelium

**Figure 2:**
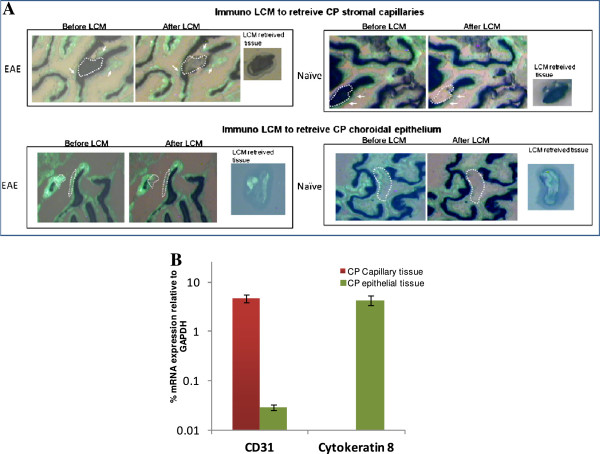
**Immuno-LCM allows retrieval of tissue from specific CP compartments.A)** Evidence of histological purity. Immunofluorescence was performed using FITC-conjugated pan-cytokeratin antibody to highlight the CP epithelium (green), while immunohistochemistry using alkaline phosphatase detection with NBT/BCIP as substrate was carried-out to label the endothelium of CP stromal capillaries (dark brown). LCM was performed on a Pixcell IIe LCM unit. Images both BEFORE and AFTER LCM, as well as LCM retrieved tissue deposited on the cap, are shown to highlight selective retrieval of CP stromal capillary (top row) and CP choroidal epithelial tissues (bottom row). **B)** Evidence of purity by qrt-PCR. Levels of CD31, an endothelial marker, and Cytokeratin-8, an epithelial marker, were probed to determine the purity of the CP capillary and CP epithelial tissues, respectively, retrieved by LCM.

Studies were next carried out to confirm the ability of LCM to resolve the stromal capillary and choroidal epithelial layers. Figure 2A shows an example of the highly selective retrieval of both tissues from naïve and EAE brain specimens. Microscopic analysis indicates no appearance of fluorescently-stained choroidal epithelial tissue in the LCM-captured capillaries and, conversely, no alkaline phosphatase-stained capillary tissue in the retrieved epithelial samples. Figure 2B further highlights the purity in qrt-PCR detection of LCM tissue from the respective CP compartments. Using equivalent amounts of input LCM tissue (1000 laser ‘shots’) from both CP compartments, the endothelial marker CD31 was significantly enriched in the CP capillary tissue, while the epithelial marker cytokeratin 8 was observed in CP epithelial tissue alone. The extremely low level of CD31 mRNA detected in CP epithelial tissue may reflect the few monocytes and/or dendritic cells circulating through this area in the steady-state mouse brain [29,30]. There is thus high confidence that LCM effectively separates CP stromal capillary from choroidal epithelial layers with high purity. 

### Expression of immune-related genes by stromal CP capillary tissue following immunization

The next series of experiments coupled LCM to TLDA qrt-PCR arrays to further characterize expression patterns of a panorama of 93 immune-related genes in the separate CP compartments at different stages of the neuroinflammatory response to immunization. Expression of these genes relative to housekeeping gene GAPDH (GAPDH was unaffected across treatments; Additional file 2: *Housekeeping control genes*), was determined in three groups of mice: MOG-CFA/PTX, CFA/PTX and naïve at two time points: day 9 and 15 p.i. Contrasting these three treatment groups enabled effects of the adjuvants CFA and PTX to be distinguished from the autoimmune response to MOG. Furthermore, examining effects at day 9 p.i. (prior to any evidence of clinical disease) and day 15 p.i. (after disease onset), highlighted the progression of gene changes that may be linked with developing pathology.

At *day 9 p.i.* (EAE clinical score 0), numerous gene changes were already evident in the CP stromal capillary tissue, despite the lack of onset of any clinical disease signs. Specifically, both MOG-CFA/PTX- and CFA/PTX-immunized mice showed up-regulated expression in 49 of the genes in the panel compared to naïve animals, (Table 1), with there being no statistically significant differences between the two immunized groups. Some prominent inflammatory genes that were equivalently elevated at this time point included: CCL2, CCL5, CXCL10, Sele (E-selectin), Selp (P-selectin), IL1b, Stat1 and Fasl all of which were modulated more than 10 fold higher than naïve levels. It would thus appear that, at this early stage before clinical EAE symptoms are present, the gene responses in the CP stromal capillary tissue following MOG immunization may stem largely from adjuvants CFA and/or PTX.

**Table 1:** Genes similarly up-regulated in CP stromal capillary tissue from both MOG-CFA/PTX- and CFA-PTX-immunized mice at day 9 p.i

**Genes modulated similarly in stromal CP capillary of MOG-CFA/PTX mice at day 9 p.i**	
**Gene name**	**Gene name**
B2m^‡^	ll15*
Bcl2l1	ll18*
C3**^‡^	ll1b**
Ccl19^‡^	ll7
Ccl2**	Lrp2
Ccl5**^‡^	Nfkb1*
Ccr2**	Nfkb2
Cd34**	Ptgs*^‡^
Cd80	Sele**
Cd86*	Selp**
Cd8a	Amad3^‡^
Col4a5	Socs2
Csf1*	Stat1**
Cxcl10**	Stat3
Cxcr3*	Stat4^‡^
Ece1	Stat6
Edn1	Tbx21
Fas	Tfrc*
Fn1	Tgfb1
Gzmb^‡^	Cd40
Hmox1	Fasl**
Hprt1	Vcam1
lcos	Vegfa
lfng**	

genes with * ≥ 5 and ** ≥ 10 fold increase in expression in comparison to Naïve animals.

Relative mRNA expression values of 93 immune-related genes were determined by immuno-LCM/TLDA in CP stromal capillary tissue from immunized and naïve mice at day 9 p.i. At this early time-point, all 49 immunization-induced genes were similarly stimulated in both MOG-CFA/PTX- and CFA-PTX-immunized mice compared to naïve animals, and only these are listed. ^‡^ denotes genes that show specific upregulation later on with disease progression on day 15.

**Figure 3:**
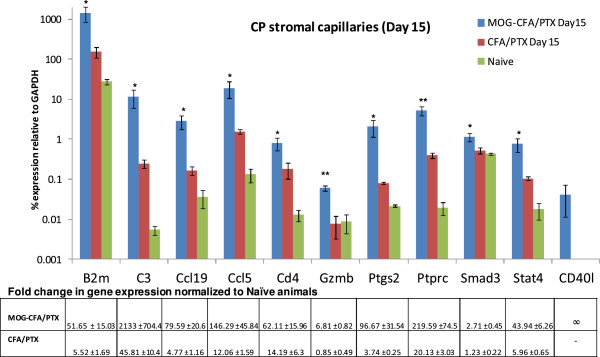
**Genes super-induced in CP stromal capillary tissue from MOG-CFA/PTX-immunized mice at day 15 p.i.** Relative mRNA expression values were determined by immuno-LCM/TLDA in CP stromal capillary tissue from MOG-CFA/PTX-immunized, CFA-PTX-immunized, and naïve mice at day 15 p.i. Those genes that were more stimulated; i.e., ‘super-induced’, in MOG-CFA/PTX- versus CFA-PTX-immunized mice at day 15 p.i. (compared to naïve mice) are graphed. The bar graphs depict those specific genes showing statistically significant differences in relative expression values (*p* values indicated by asterisks) between MOG-CFA/PTX and CFA/PTX experimental groups. RNA values are presented as mean percent expression relative to GAPDH (± SEM) in log scale. * and ** represent comparisons made between MOG-CFA/PTX and CFA-PTX- treatment groups, * p < 0.05, ** p < 0.005, Student’s *t*-test, n = 3 animals/group. Fold changes in gene expression normalized to naïve animals are tabulated below the graph. Fold changes of those genes that were up-regulated after immunizations but undetectable in naïve are denoted as ‘∞’.

By *day 15 p.i.* (EAE clinical score 1.5-2.0), however, the MOG-CFA/PTX-immunized group surpassed the CFA/PTX group in up-regulation of several genes, highlighting what might specifically be the autoimmune response of the CP vascular stroma. These genes included B2m, C3, CCL19, CCL5, CD4, Gzmb, Ptgs2, Ptprc (CD45), Smad3, Stat4, and CD40l – which were selectively augmented in the CP stromal capillary tissue of the MOG-CFA/PTX group (Figure 3). The fold changes in these genes following immunization (Figure 3, bottom) indicate their super-stimulation by MOG-CFA/PTX treatment. The lymphoid chemokine CCL19, expressed by venules in brain and spinal cord in mice afflicted with EAE [31], was elevated nearly 80-fold in the MOG-CFA/PTX immunized mice at this time-point. And CCL5, another chemokine shown to play an important role in EAE [32], was elevated 146-fold higher than naïve levels. Expression of Ptgs2 (COX2), suppression of which has been associated with resistance to EAE [33,34], was near similarly elevated – having increased 96-fold higher than that in naïve cohorts. Slightly less elevated was Stat4, a transcription factor whose absence has been shown to inhibit EAE [35], which was increased > 40-fold in the MOG immunized group. It is further noteworthy that expression of Ptprc (CD45), the common leukocyte marker, stimulated 219-fold higher, possibly reflecting increased leukocyte extravasation across the stromal capillaries at this later time-point. Consistent with this interpretation is that message for CD40l (CD154), a protein primarily expressed on activated T cells [36], was detected within the CP stromal capillary tissue only following MOG-CFA/PTX immunization. 

Notably, the CFA/PTX group indicated some dampening of immune regulation by this time, as certain genes e.g., Smad3 and Gzmb, dropped back to levels matching those of naïve mice, after initially displaying an elevation at day 9. Genes that trended towards elevated expression following MOG-CFA/PTX treatment (but with *p* values slightly > 0.05) are displayed in Additional file 3: *Genes that trended towards elevated expression in MOG-CFA/PTX- immunized CP stromal capillary tissue compared to CFA-PTX-immunized mice, at day 15 p.i*, while those that were similarly up-regulated in CP stromal capillary tissue of MOG-CFA/PTX- and CFA/PTX-immunized mice compared to naïve mice at day 15 are listed in Additional file 4: *Genes similarly up-regulated in CP stromal capillary tissue from both MOG-CFA/PTX- and CFA-PTX-immunized mice at day 15 p.i.* Genes that were undetected in the CP capillary tissue in all treated and naïve mice at both time-points were the following: CCR4, CD19, CD3e, CSF3, Ctla4, Cyp1a2, Cyp7a1, H2-Ea, IL12b, IL13, IL3, IL4, IL5, IL6, IL9, and Lta.

### Expression of immune-related genes by CP choroidal epithelium following immunization

Immunization also produced a change in expression of numerous immune-related genes within the CP choroidal epithelium. Moreover, these changes differed from those observed in the capillary stroma, emphasizing the differential immune sensitivities of the two tissues.

**Figure 4:**
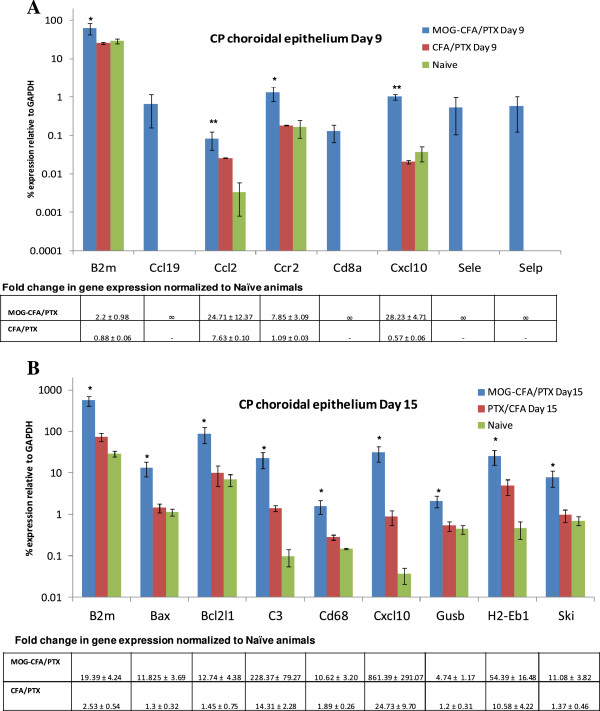
**Genes super-induced in CP choroidal epithelial tissue from MOG-CFA/PTX-immunized mice at days 9 and 15 p.i.** Relative mRNA expression values of 93 immune-related genes were determined by immuno-LCM/TLDA in CP choroidal epithelial tissue from MOG-CFA/PTX-immunized, CFA-PTX-immunized, and naïve mice at two time-points. Those genes that were more stimulated in MOG-CFA/PTX- versus CFA-PTX-immunized mice at day 9 (Figure 4**A**) and day 15 p.i (Figure 4**B**) (compared to naïve mice) are graphed. The bar graphs depict those specific genes showing statistically significant differences in relative expression values (*p* values indicated by asterisks) between MOG-CFA/PTX and CFA/PTX experimental groups. RNA values are presented as mean percent expression relative to GAPDH (± SEM) in log scale. * and ** represent comparisons made between MOG-CFA/PTX and CFA-PTX- treatment groups, * p < 0.05, ** p < 0.005, Student’s *t*-test, n = 3 animals/group. Fold changes in gene expression normalized to naïve animals are tabulated below the corresponding graphs. Fold changes of those genes that were up-regulated after immunizations but undetectable in naïve are denoted as ‘∞’.

At *day 9 p.i.,* the CP choroidal epithelium of only MOG-CFA/PTX-immunized mice displayed increased expression of any immune-related genes compared to that of naïve cohorts. Specifically, the following eight immune-related genes were up-regulated: B2m, CCL19, CCL2, CCR2, CD8a, CXCL10, Sele, and Selp (Figure 4A). B2m (beta 2 microglobulin), a biomarker for certain peripheral inflammatory conditions [37] was increased 2.2-fold in MOG-treated versus naïve mice. The chemokine CCL2 has been demonstrated to play a critical, non-redundant role in directing mononuclear leukocyte extravasation into the CNS during EAE [32,38,39], and was stimulated > 24-fold higher in the CP choroidal epithelial tissue of MOG-CFA/PTX-treated mice compared to that in naïve cohorts. CXCL10 and CCL19 were 28-and 14-fold higher than naïve values, respectively. Sele (E-selectin) and Selp (P-selectin), CCL19 and CD8a further showed pronounced stimulation specifically following MOG immunization, being undetectable in the epithelium of both CFA/PTX and naïve cohorts. In what appears to reflect the differential sensitivities of the two CP tissues, CFA/PTX immunization clearly ‘activated’ the stromal CP capillary tissue on day 9 p.i. at both the anatomical and molecular levels (Figure 1 and Table 1), but produced no detectable changes in the CP epithelium at this time. 

By *day 15 p.i.,* genes B2m and CXCL10 displayed further increases in expression in the CP choroidal epithelium of MOG-CFA/PTX mice compared to that seen in this cohort at day 9 (Figure 4B), showing > 19-fold and > 800-fold higher levels, respectively, compared to naïve mice. Expression levels of yet additional genes in MOG-treated mice also became elevated by this time; these included Bax, Bcl2l1, C3, CD68, Gusb, H2-Eb1 and Ski (Figure 4B). Moreover, genes CCL19, CCL2, CCR2, CD8a, Sele and Selp, which had previously shown up-regulation only in the MOG-CFA/PTX group at day 9, became similarly induced in the CFA/PTX group at this later time-point. Genes that trended towards elevated expression following MOG-CFA/PTX treatment for both time points (but with *p* values slightly >0.05) are displayed in Additional file 5 and Additional file 6: *Genes that trended towards elevated expression in MOG-CFA/PTX immunized CP epithelium tissue compared to CFA-PTX-immunized mice, at day 9/15p.i.,* while those that were similarly up-regulated in CP choroidal epithelial tissue of MOG-CFA/PTX- and CFA/PTX-immunized mice compared to naïve mice for both time points are listed in Additional file 7 and Additional file 8: *Genes similarly up-regulated in CP epithelium from both MOG-CFA/PTX- and CFA-PTX-immunized mice at day 9/15 p.i.* Those few genes that were in the CP epithelial tissue in both immunized groups and naïve mice included IL3, IL4, IL5, IL6, Lta.

### Interaction between immunization treatment and time

**Figure 5:**
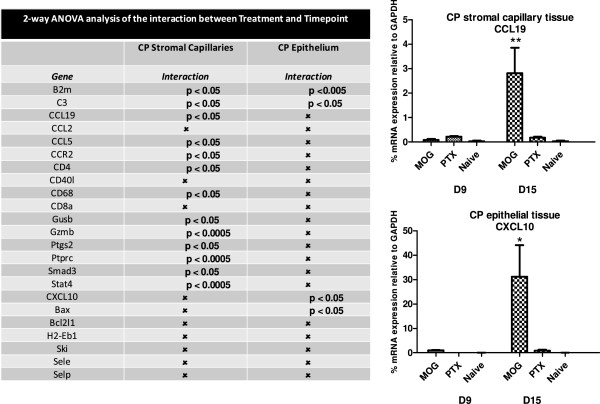
**Interaction between immunization treatment and time of analysis post-immunization on expression of immune-related genes within CP stromal capillary and choroidal epithelial compartments.** Interactive effects of treatment (MOG-CFA/PTX, CFA-PTX and naïve) and time post-immunization were determined by two-way ANOVA on the 23 immune-related genes that were super-induced following MOG-CFA/PTX immunization in either of the two CP compartments (those genes graphed in Figures 3, 4A and B). Of these 23 genes, 13 genes in the CP stromal capillary and 4 genes in the CP epithelial tissue exhibited significant *positive interaction* between treatment and time post-immunization, and are denoted with their corresponding *p* values. The remaining genes that showed no significant interaction are labeled by (*x*). Time-dependent changes in expression of CCL19 in stromal capillary tissue and CXCL10 in choroidal epithelial tissue are graphed as representative examples * *p* < 0.05, ** *p* < 0.005.

In order to gain further appreciation of the extent to which time impacted the effect of specific type immunization on the expression patterns of immune-related genes, two-way ANOVA was performed to deduce *interactive effects* between immunization treatment (e.g., MOG-CFA/PTX, CFA/PTX or naïve) and time post-immunization. For example, the expression of CCL19 in CP stromal capillary tissue following MOG-CFA/PTX immunization was time-dependent (*p* < 0.05 for positive interaction). Two-way ANOVA was done on all genes that displayed statistically significant modulation after immunization in at least one of the CP compartments (as shown in Figures 3 and 4A, B) in either of the time-points analyzed (twenty-three genes in total). Interactive effects differed depending on the CP compartment, further highlighting the unique responses of the two CP tissues analyzed. Specifically, two-way ANOVA of CP stromal capillary tissue revealed the following twelve genes displayed *positive interaction* between immunization treatment and time post-immunization: B2m, C3, CCL19, CCL5, CD4, Gzmb, Ptgs2, Ptprc, Stat4, CCR2, CD68, Gusb (Figure 5). CP choroidal epithelial tissue, on the other hand, demonstrated *positive interaction* for another collective of genes: B2m, Bax, C3, CXCL10 (Figure 5).

**Figure 6:**
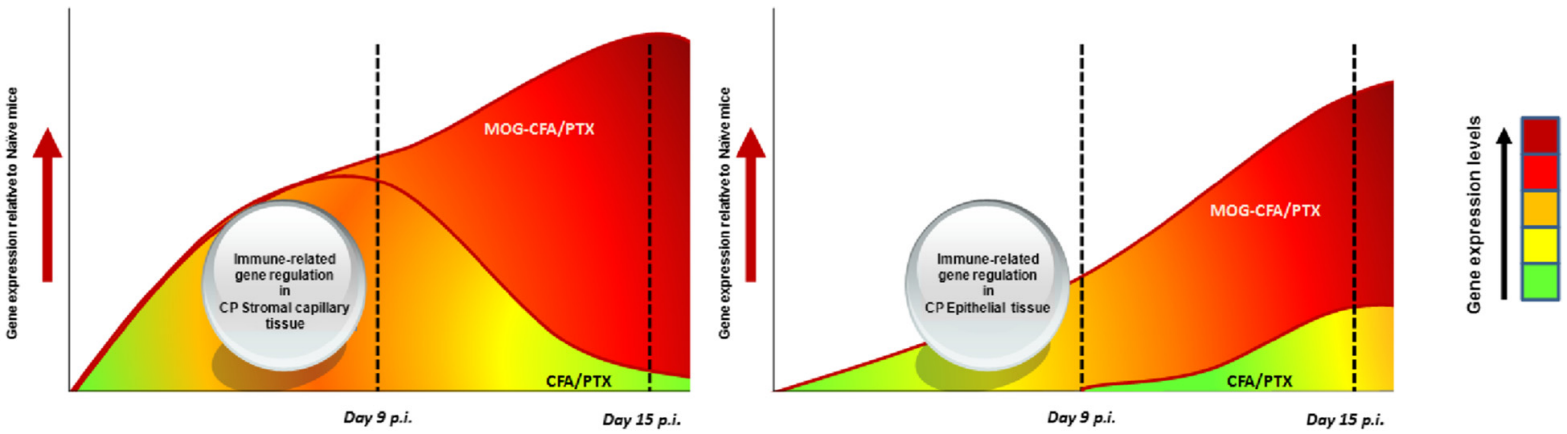
**Schematic summary of the induction of immune-related genes in the choroid plexus compartments following MOG-CFA/PTX versus CFA-PTX immunization.** Depicted are qualitative ‘heat maps’ of overall modulation in immune-related gene expression patterns over time, within the separate CP stromal capillary (left) and CP choroidal epithelial (right) tissues after the two type immunization regimens. While CFA/PTX immunization alone induces expression of immune-related genes in both tissues, MOG-CFA/PTX immunization causes supernumerary stimulation above this level. The differences in gene expression between the immunization regimens highlight what might reflect gene changes specifically due to MOG-associated autoimmunity. The effects due solely to CFA/PTX immunization might reveal priming of the immune response that is necessary for the development of EAE.

Figure 6 qualitatively summarizes the differential responses of the CP capillary and CP choroidal epithelial tissues, respectively, to MOG-CFA/PTX immunization versus CFA/PTX immunization, contrasting adjuvant versus autoimmune effects on immune-related gene regulation over the two time-points analyzed.

## Discussion

Due to increasing awareness of the CP as fundamental to the development of CNS inflammation [4-6], immuno-LCM coupled to qrt-PCR array was used to separately acquire CP stromal capillary and choroidal epithelial tissues and assess their respective patterns of expression *in situ* of a wide panorama of immune-related genes. Gene patterns were evaluated during preclinical and early clinical stages of EAE to appreciate the switches in gene expression that accompany evolving disease.

It is clear that the CP responds vigorously to MOG immunization at both the anatomical and molecular levels. Interestingly, immunization with CFA and PTX alone produced striking effects. Swelling of the capillary plexus occurred to nearly the same extent with injection of just these agents, as with PTX and MOG in CFA. PTX is an ancillary adjuvant commonly employed to elicit EAE, as well as several other experimental autoimmune diseases [40-44]. And while its mechanism of action in this regard has generally been attributed to increasing vascular permeability [45-47] – most notably that of the BBB [48-51] – additional hypotheses have been put forth [52-55]. However, to the best of our knowledge, this is the first report to turn attention to the CP as a possible target of PTX. It is of further interest to point out that the distension of CP capillaries noted here study bears similarity to that seen following systemic neutralization of VEGF and TGFβ [56]. In the latter case, CP capillary swelling was accompanied by loss of fenestrae from endothelial cells and appearance of multiple caveolae, transport vesicles that transcytose a variety of cargo [57] – including chemokines [58] – and are often associated with heightened vascular permeability and inflammation [59,60]. Engelhardt et al. [4] had also described ultrastructural changes of the CP during EAE (along with CFA and PTX as adjuvants), but noted these were mostly restricted to the CP choroidal epithelium. Moreover, as comparison in this latter study was just between healthy mice and those afflicted with EAE, it is unclear whether the observed epithelial response was autoimmune in nature and/or due to adjuvant action.

Our results suggest that injection of PTX and/or CFA, alone, might trigger an immune response in the CP capillaries that helps “set the stage” for CNS inflammation [61]. The CP capillaries might be uniquely responsive in this regard, as CFA/PTX treatment evoked an early response (day 9 p.i.) in the CP stromal capillary tissue, while the choroidal epithelium experienced neither overt morphological nor gene expression changes at this time. If, as speculated during MS/EAE, Th17 cells first transit through the CP, and then travel in the CSF to reach their cognate antigens in the SAS, then the CP capillaries must somehow initially be rendered capable of supporting T cell extravasation. In the EAE paradigm used here, PTX and/or CFA might provide the stimulus to evoke such capability. In this regard, we noted > 10-fold increase in chemokines CCL2, CCL5 and CXCL10 in the CP stromal capillaries of both MOG-CFA/PTX- and CFA/PTX-treated mice at day 9 p.i. Constitutive CCL2 expression within the CP stromal tissue has been reported using *in situ* hybridization analysis, and shown to be induced following peripheral tissue inflammation [62]. The ability of CFA/PTX treatment to stimulate expression of these chemokines could potentially reflect the actions of one or both of these adjuvants to ‘prime’ the neuroinflammatory process by activating the endothelium to elicit initial auto reactive T cell extravasation from the circulation into the stromal compartment. This hypothesis is consistent with the recent observation that administration of PTX to transgenic mice over-expressing CCL2 in the CNS causes disruption of the BBB and promotes leukocyte migration into the brain parenchyma [63].

Notwithstanding the effects of CFA/PTX treatment on CP capillary morphology and gene expression, immunization with MOG-CFA/PTX further induced the expression of additional genes – some or all of which might specifically reflect the autoimmune response and associated development of EAE. While perhaps necessary for disease to develop, the CP conditions set in place by PTX and CFA are insufficient for inducing EAE in wild-type C57BL/6 mice. For disease to occur, supernumerary induction of some genes, and *de novo* induction of others must take place. The findings by Goverman et al. and Brabb et al. [61,64], that injection of PTX alone can “trigger” EAE in TCR-transgenic mice specific for myelin basic protein, by fostering T cell access to the CNS, comports with our results and the view that PTX enables mice to reach the disease threshold. And helping pull this trigger may be additional effects of PTX on T cell behavior. Our observation of increased mRNA for genes CD8a, CD80, CD86, Gzmb (granzyme) and Ptprc (CD45) in the CP capillary stromal tissue of both MOG-CFA/PTX and CFA/PTX cohorts may reflect capture of PTX-activated CD8 T cells in transit across the CP and into the CSF. This interpretation is consistent with the recent finding by Murphey et al. [65], that PTX stimulation of cultured spleen cells results in CD 8 T cell activation via CD80/86 co-stimulation.

As to signals responsible for the extravasation of T cells from the circulation into the CP stroma during MOG-induced EAE, a combination of chemokines may fill this role, as these immune mediators do in guiding parenchymal leukocyte infiltration. In particular, CCL5 level was increased significantly in CP stromal capillary tissue at day 15 p.i., which coincides with high CCL5 protein level reported in whole brain extract of EAE mice at a similar time-point, and argued to mediate leukocyte adherence to the CNS microvasculature [32]. And CCL19 – a CCR7 ligand known to play a crucial role in EAE development through IL-23 producing Th17 cells [66] – was likewise up-regulated dramatically in the CP stromal capillaries of MOG immunized mice at day 15 p.i.

With specific regard to those mechanism(s) further driving T cell migration from the CP stroma into the CSF, recent evidence points toward expression of another chemokine - CCL20 - by the CP choroidal epithelium as directing CCR6^+^ T cells across this layer and into ventricular fluid during MS/EAE [8]. However, as this chemokine:cognate receptor pair was not represented on the commercial TLDA card used in these experiments, confirmation of this pathway was not performed. Aside from CCL20 providing a driving force for T cell migration into CP epithelium, CCL2 might also serve in this capacity, as Chodobska et al. [67] noted the latter chemokine was rapidly stimulated in this tissue *in vivo* and then released into the CSF, following traumatic brain injury. Indeed, the significant increase in CCL2 we observed in the MOG-CFA/PTX CP choroidal epithelium at day 9 p.i. might just reflect such a role for this chemokine in EAE. The cognate receptor for CCL2, CCR2 was also seen to be elevated in the choroidal epithelium at this early time-point. Presently, it is unclear if the high levels of CCR2 mRNA indicate activation of the epithelium, which, in the periphery, has been shown to express CCR2 [68,69], or the accumulation of infiltrating CCR2^+^ T cells.

In what might suggest multi-level control of leukocyte extravasation into the CSF, still other chemokines were also significantly up-regulated by the CP during EAE – namely CXCL10 and CCL19. As CXCL10 has been reported to be up-regulated in the sub-ventricular zone (SVZ) during EAE, and postulated to stimulate migration of activated T cells into the SVZ [70], its spike in expression by CP choroidal epithelial tissue at day 9 p.i. and more robust elevation by day 15 p.i., might imply this chemokine is obligate for T cell entry into the ventricles. In analogous manner, CCL19 was also elevated at this site at day 9 p.i. In fact, the timing of the CP epithelial spikes in this chemokine during early stages of EAE noted here, coincides well with that reported by Reboldi et al. [8] for initial T cell entry into the uninflamed CNS through the CP. Recently, Marques et al. [22,71] used hybridization-based microarray to assess the global transcriptome of the whole CP following chronic peripheral LPS stimulation. When compared to our study, there were some common and unique findings. Among the common findings, complement protein C3, and chemokines CCL2 and CCL5 were elevated following either *acute* or *chronic* peripheral LPS stimulation, as well as during MOG-induced EAE (complement C3 in both CP capillary tissue and epithelium; CCL2 in CP epithelium; and CCL5 in CP capillary tissue). And Selectin (Sele and Selp) expression was also elevated both following *acute* peripheral LPS stimulation [22], and in the CP epithelium after MOG-induced EAE. These common gene modulations may thus reflect more generic CP inflammatory response genes. As for unique findings, these too involved chemokines. Marques et al. [71] reported stimulation of CCL7 and CXCL1 in the CP following *chronic* LPS stimulation, while we detected stimulation of CCL19 and CXCL10 in the CP epithelium and CCL19 in the CP endothelium during MOG-induced EAE. *A priori*, up-regulation of these latter two chemokine genes may more distinguish an EAE signature for the respective CP tissue compartments.

Of further note was our observation of a MOG-sensitive increase in expression of B2m at day 15 p.i. in CP stromal capillary tissue, and at both time-points in the choroidal epithelial tissue. Aside from perhaps reinforcing a more ubiquitous role for B2m in inflammation [37], this result complements previous reports of increased B2m mRNA and protein levels in both neuronal and non neuronal cell types during EAE [72,73], a response thought to be due to induction of synaptic plasticity by infiltrating autoreactive immune cells. The sharp induction of complement C3, also noted in both CP compartments of MOG-CFA/PTX mice at the later time point (day 15 p.i), further supports previous studies highlighting C3 deficiency inhibits development of EAE [74].

As to the specific approach used here, i.e., LCM coupled to TLDA, it offered extraordinary opportunity to probe, in extensive detail, the focused immune response within the distinct CP compartments. Earlier reports, using immunohistochemistry and *in situ* hybridization, had shown that the respective CP stromal capillary endothelial cells and the CP choroidal epithelial cells displayed different expression patterns of a small nucleus of adhesion molecules during EAE [4,20]. Specifically, VCAM-1 and ICAM-1 were found to be expressed constitutively by CP choroidal epithelial cells of healthy SJL/N mice, and then further induced following active immunization with spinal cord homogenate. MAdCAM-1 was only seen in these cells after induction of EAE. However, none of these adhesion molecules, nor E- or P-selectin, was detected in CP stromal capillary endothelial cells [75].

We too noted constitutive VCAM-1 expression in the CP choroidal epithelial tissue of healthy naïve mice. Likewise, VCAM-1 trended toward elevation within this CP compartment of MOG-CFA/PTX cohorts at both days 9 and 15 p.i., though it showed no up-regulation in CFA-PTX-immunized mice at either time-point. In further agreement with previous observations [75], our analysis demonstrated induction of E-selectin and P-selectin in the CP choroidal epithelial tissue from MOG-CFA/PTX-immunized mice at day 9. Our results nevertheless displayed some stark differences with earlier reports. Specifically, we also noted a trend of increased VCAM-1 expression by day 15 in CP stromal capillary tissue with MOG immunization, paralleling what has been described in MS brain tissue [6]. And both E- and P-selectin mRNA were also observed to be induced in CP stromal capillary tissue of both MOG-CFA/PTX- and CFA/PTX-immunized mice compared to that of naïve mice at day 9 p.i. E-selectin increased expression in the two immunized groups by >100-fold, while P-selectin was stimulated >10-fold.

*A priori*, differences in results between these EAE studies could result from several factors, among them being 1) the EAE model employed (e.g., immunization of SJL/6 mice with spinal cord homogenate versus immunization of C57BL/6 mice with MOG_35-55_ peptide), the time of analysis post-immunization (e.g, before or after disease onset), and 3) the sensitivity of the analytic techniques (e.g., *in situ* hybridization versus qrt-PCR). As neither MAdCAM-1 nor ICAM-1 were represented on the TLDA card used in these experiments, confirmation of expression or lack thereof was not possible for these genes.

Most recently, Liddelow et al. [76] employed LCM to collect mouse lateral ventricular CP tissue CP for transcriptome analysis of transporter gene expression during normal development. Here, we extended this application, utilizing LCM to resolve – for the first time – the CP capillary stromal tissue from the CP choroidal tissue, and then separately analyzing each for their unique immune responses to MOG immunization.

## Conclusions

Induction of EAE in C57BL/6 mice by active immunization with MOG_35-55_ peptide results in the respective CP stromal capillary and choroidal epithelial compartments each mounting vigorous, yet distinct, immune responses, underscoring the active role of the CP in instigating CNS inflammatory disease. Furthermore, our results make clear that a significant component of the total CP response is due to effects elicited by adjuvants PTX and/or CFA used in the immunization protocol – which might serve to prime the CP to support autoimmune activity necessary for developing MS/EAE. These results are summarized schematically in Figure 6.

## Competing interests

The authors have no competing interests.

## Authors’ contributions

N. Murugesan assisted in the design of the experiments, developed the immuno-LCM protocol for evaluating the different CP tissues, performed the immuno-LCM/TLDA analyses of CP tissues and microscopic evaluation of CP structure in response to immunization, and contributed to the writing and editing of the manuscript. D. Paul assisted with the 3-D image analysis of CP structure. B. Shrestha assisted with the immuno-LCM/TLDA analyses. Y. Lemire and S. Ge assisted with the immunizations. J. Pachter designed the experiments, wrote the manuscript and provided oversight for all studies. All authors have read and approved the final version of the manuscript.

## Supplementary Material

Additional file 1 **Mouse Immune Panel TLDA.** The card map for the 96 genes (93 immune-related genes and 3 control genes) on the commercially available mouse Immune panel TLDA is shown, with gene names and corresponding accession numbers. Click here for file

Additional file 2 **Housekeeping control genes.** Ct (Threshold cycle) values for the three housekeeping genes – GAPDH, β-Actin and 18 S represented on the mouse Immune-panel TLDA are shown. The housekeeping genes were almost unchanged across treatments (shown in A and B) with < 1 cycle difference between samples. C, Six genes were normalized to each of the three housekeeping gene and expression patterns plotted, indicating identical patterns of expression across housekeeping control gene used. Click here for file

Additional file 3 **Genes that trended towards elevated expression in MOG-CFA/PTX- immunized CP stromal capillary tissue compared to CFA-PTX-immunized mice, at day 15 p.i*.*** Relative mRNA expression values of 93 immune-related genes were determined by immuno-LCM/TLDA in CP stromal capillary tissue from immunized and naïve mice at day 15 p.i. A total of 14 genes trended towards greater induction in the MOG-CFA/PTX group compared to the CFA-PTX group; these genes are listed with their corresponding *p* values. Analysis was by Student’s two-tailed *t*-test. Click here for file

Additional file 4 **Genes similarly up-regulated in CP stromal capillary tissue from both MOG-CFA/PTX- and CFA-PTX-immunized mice at day 15 p.i*.*** Relative mRNA expression values of 93 immune-related genes were determined by immuno-LCM/TLDA in CP stromal capillary tissue from immunized and naïve mice at day 15 p.i. At this later time-point, 25 immunization-induced genes were similarly stimulated in both MOG-CFA/PTX- and CFA-PTX-immunized mice compared to naïve animals, and only these are listed. Click here for file

Additional file 5 **Genes that trended towards elevated expression in MOG-CFA/PTX immunized CP epithelium tissue compared to CFA-PTX-immunized mice, at day 9 p.i*.*** Relative mRNA expression values of 93 immune-related genes were determined by immuno-LCM/TLDA in CP epithelium from immunized and naïve mice at day 9 p.i. A total of 15 genes trended towards greater induction in the MOG-CFA/PTX group compared to the CFA-PTX group; these genes are listed with their corresponding *p* values. Analysis was by Student’s two-tailed *t*-test. Click here for file

Additional file 6 **Genes that trended towards elevated expression in MOG-CFA/PTX immunized CP epithelium tissue compared to CFA-PTX-immunized mice, at day 15 p.i*.*** Relative mRNA expression values of 93 immune-related genes were determined by immuno-LCM/TLDA in CP epithelium from immunized and naïve mice at day 15 p.i. A total of 19 genes trended towards greater induction in the MOG-CFA/PTX group compared to the CFA-PTX group; these genes are listed with their corresponding *p* values. Analysis was by Student’s two-tailed *t*-test. Click here for file

Additional file 7 **Genes similarly up-regulated in CP epithelium from both MOG-CFA/PTX- and CFA-PTX-immunized mice at day 9 p.i*.*** Relative mRNA expression values of 93 immune-related genes were determined by immuno-LCM/TLDA in CP epithelium from immunized and naïve mice at day 9 p.i. At this early time-point, 10 immunization-induced genes were similarly stimulated in both MOG-CFA/PTX- and CFA-PTX-immunized mice compared to naïve animals, and only these are listed. Click here for file

Additional file 8 **Genes similarly up-regulated in CP epithelium from both MOG-CFA/PTX- and CFA-PTX-immunized mice at day 15 p.i*.*** Relative mRNA expression values of 93 immune-related genes were determined by immuno-LCM/TLDA in CP epithelium from immunized and naïve mice at day 15 p.i. At this later time-point, 8 immunization-induced genes were similarly stimulated in both MOG-CFA/PTX- and CFA-PTX-immunized mice compared to naïve animals, and only these are listed. Click here for file
