# Supplementary figures and images for: Active induction of experimental autoimmune encephalomyelitis by MOG35-55 peptide immunization is associated with differential responses in separate compartments of the choroid plexus
Source: Fluids Barriers CNS. 2012 Aug 7;9:15. doi: 10.1186/2045-8118-9-15 (PMC3493354; doi:10.1186/2045-8118-9-15)

## Additional file 2

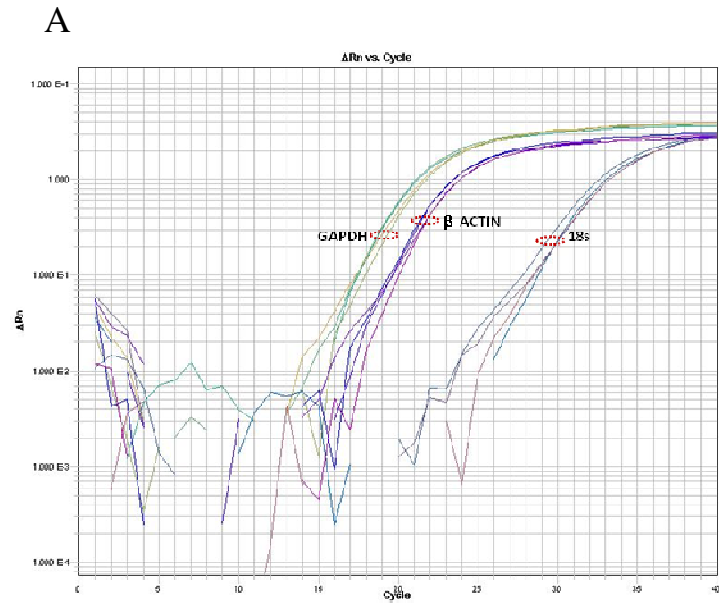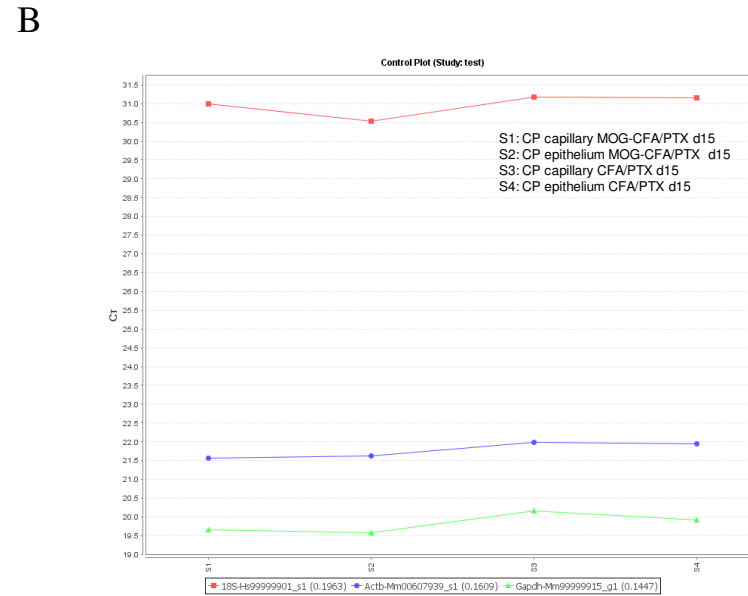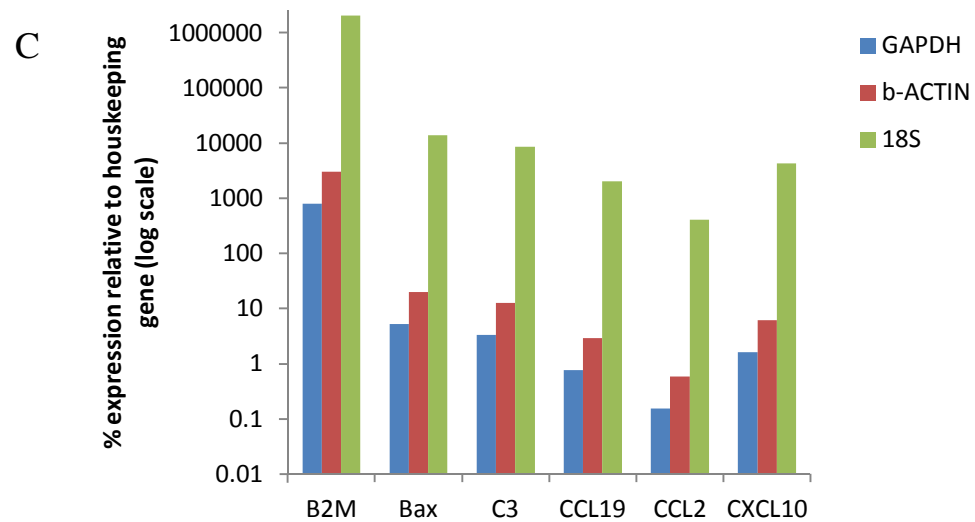

Supplement: Additional file 2 — Housekeeping control genes. Ct (Threshold cycle) values for the three housekeeping genes – GAPDH, β-Actin and 18 S represented on the mouse Immune-panel TLDA are shown. The housekeeping genes were almost unchanged across treatments (shown in A and B) with < 1 cycle difference between samples. C, Six genes were normalized to each of the three housekeeping gene and expression patterns plotted, indicating identical patterns of expression across housekeeping control gene used. [file 2045-8118-9-15-S2.pdf]
