# Supplementary material for: Active induction of experimental autoimmune encephalomyelitis by MOG35-55 peptide immunization is associated with differential responses in separate compartments of the choroid plexus
Source: Fluids Barriers CNS. 2012 Aug 7;9:15. doi: 10.1186/2045-8118-9-15 (PMC3493354; doi:10.1186/2045-8118-9-15)
Supplement: Additional file 3 — Genes that trended towards elevated expression in MOG-CFA/PTX- immunized CP stromal capillary tissue compared to CFA-PTX-immunized mice, at day 15 p.i. Relative mRNA expression values of 93 immune-related genes were determined by immuno-LCM/TLDA in CP stromal capillary tissue from immunized and naïve mice at day 15 p.i. A total of 14 genes trended towards greater induction in the MOG-CFA/PTX group compared to the CFA-PTX group; these genes are listed with their corresponding p values. Analysis was by Student’s two-tailed t-test. [file 2045-8118-9-15-S3.pdf]

Additional file 3

Gene	MOG-CFA/PTX CP Stromal capillaries D15<br><br>Avg % expn. ±sem	CFA/PTX CP Stromal capillaries D15<br><br>Avg % expn. ±sem	*p* value
**Bax**	31.79 ± 12.62	3.54 ± 0.89	0.081044
**Ccr2**	8.57 ± 3.26	1.18 ± 0.86	0.077816
**Cd68**	0.83 ± 0.22	0.32 ± 0.066	0.075827
**Csf1**	12.00 ± 4.4	1.82 ± 0.65	0.074886
**Cxcl10**	18.20 ± 9.31	0.87 ± 0.21	0.129095
**Edn1**	2.99 ± 1.31	0.35 ± 0.09	0.10678
**Fn1**	19.42 ± 8.42	1.55 ± 0.12	0.093156
**Gusb**	5.94 ± 2.02	1.15 ± 0.09	0.068766
**Nfkb2**	6.42 ± 2.58	0.22 ± 0.08	0.06632
**Ski**	14.44 ± 5.81	2.77 ± 0.65	0.108814
**Stat1**	5.68 ±1.98	1.12 ±0.26	0.076147
**Tfrc**	41.32 ±18.46	4.36 ± 2.12	0.109386
**Vcam1**	27.79 ± 8.66	9.39 ±3.85	0.108927
**Vegfa**	14.39 ±5.06	3.53 ± 1.08	0.095283
