# Supplementary material for: Active induction of experimental autoimmune encephalomyelitis by MOG35-55 peptide immunization is associated with differential responses in separate compartments of the choroid plexus
Source: Fluids Barriers CNS. 2012 Aug 7;9:15. doi: 10.1186/2045-8118-9-15 (PMC3493354; doi:10.1186/2045-8118-9-15)
Supplement: Additional file 4 — Genes similarly up-regulated in CP stromal capillary tissue from both MOG-CFA/PTX- and CFA-PTX-immunized mice at day 15 p.i. Relative mRNA expression values of 93 immune-related genes were determined by immuno-LCM/TLDA in CP stromal capillary tissue from immunized and naïve mice at day 15 p.i. At this later time-point, 25 immunization-induced genes were similarly stimulated in both MOG-CFA/PTX- and CFA-PTX-immunized mice compared to naïve animals, and only these are listed. [file 2045-8118-9-15-S4.pdf]

Additional file 4

Genes modulated similarly in CP stromal capillary of MOG-CFA/PTX and CFA/PTX at day 15 p.i.	
Gene name	Gene name
Agtr2	Hmox1
Bcl2	Hprt1
Bcl2l1	Icos
Ccl2	Il15
Cd28	Il18
Cd34	Il1b
Cd38	Lrp2
Cd86	Nfkb1
Cd8a	Nos2
Col4a5	Tgfb1
Cxcl11	Tnf
Cxcr3	Fasl
H2-Eb1	
