# Supplementary material for: Active induction of experimental autoimmune encephalomyelitis by MOG35-55 peptide immunization is associated with differential responses in separate compartments of the choroid plexus
Source: Fluids Barriers CNS. 2012 Aug 7;9:15. doi: 10.1186/2045-8118-9-15 (PMC3493354; doi:10.1186/2045-8118-9-15)
Supplement: Additional file 5 — Genes that trended towards elevated expression in MOG-CFA/PTX immunized CP epithelium tissue compared to CFA-PTX-immunized mice, at day 9 p.i. Relative mRNA expression values of 93 immune-related genes were determined by immuno-LCM/TLDA in CP epithelium from immunized and naïve mice at day 9 p.i. A total of 15 genes trended towards greater induction in the MOG-CFA/PTX group compared to the CFA-PTX group; these genes are listed with their corresponding p values. Analysis was by Student’s two-tailed t-test. [file 2045-8118-9-15-S5.pdf]

Additional file 5

Gene	MOG-CFA/PTX CP Epithelium D9 Avg % expn. ±sem	CFA/PTX CP Epithelium D9 Avg % expn. ±sem	*p* value
**C3**	1.10 ± 0.63	0.03 ± 0.002	0.098973
**Cd4**	0.08 ± 0.03	0.02 ± 0.0009	0.071996
**Cd86**	0.57 ± 0.34	0.10 ± 0.0023	0.156702
**Csf1**	0.53 ± 0.07	0.42 ± 0.004	0.101749
**Edn1**	0.23 ± 0.15	0.01 ± 0.00006	0.147027
**Fas**	0.13 ± 0.05	0.05 ± 0.001	0.07602
**Gzmb**	0.04 ± 0.02	0.003 ± 0.0002	0.118426
**H2-Eb1**	5.69 ± 3.17	0.38 ±0.02	0.101352
**Il1b**	0.28 ± 0.14	0.06 ± 0.007	0.109545
**Lrp2**	1.02 ± 0.34	0.37 ± 0.01	0.070257
**Ptgs2**	0.12 ± 0.06	0.006 ± 0.0002	0.066443
**Ski**	1.19 ± 0.26	0.73 ± 0.03	0.090216
**Stat1**	1.65 ± 0.60	0.80 ± 0.02	0.153239
**Tgfb1**	0.92 ± 0.47	0.20 ± 0.001	0.126723
**Vcam1**	7.12 ± 3.31	2.89 ± 0.10	0.187232
