# Supplementary material for: Active induction of experimental autoimmune encephalomyelitis by MOG35-55 peptide immunization is associated with differential responses in separate compartments of the choroid plexus
Source: Fluids Barriers CNS. 2012 Aug 7;9:15. doi: 10.1186/2045-8118-9-15 (PMC3493354; doi:10.1186/2045-8118-9-15)
Supplement: Additional file 6 — Genes that trended towards elevated expression in MOG-CFA/PTX immunized CP epithelium tissue compared to CFA-PTX-immunized mice, at day 15 p.i. Relative mRNA expression values of 93 immune-related genes were determined by immuno-LCM/TLDA in CP epithelium from immunized and naïve mice at day 15 p.i. A total of 19 genes trended towards greater induction in the MOG-CFA/PTX group compared to the CFA-PTX group; these genes are listed with their corresponding p values. Analysis was by Student’s two-tailed t-test. [file 2045-8118-9-15-S6.pdf]

Additional file 6

Genes modulated similarly in CP Epithelium of MOG-CFA/PTX and CFA/PTX at day 9 p.i.	
Gene name	Gene name
Bcl2l1	Nfkb1
Ccl3	Pgk1
Ccl5	Stat4
Cd68	Tfrc
Hprt1	Fasl
