# Supplementary material for: Active induction of experimental autoimmune encephalomyelitis by MOG35-55 peptide immunization is associated with differential responses in separate compartments of the choroid plexus
Source: Fluids Barriers CNS. 2012 Aug 7;9:15. doi: 10.1186/2045-8118-9-15 (PMC3493354; doi:10.1186/2045-8118-9-15)
Supplement: Additional file 7 — Genes similarly up-regulated in CP epithelium from both MOG-CFA/PTX- and CFA-PTX-immunized mice at day 9 p.i. Relative mRNA expression values of 93 immune-related genes were determined by immuno-LCM/TLDA in CP epithelium from immunized and naïve mice at day 9 p.i. At this early time-point, 10 immunization-induced genes were similarly stimulated in both MOG-CFA/PTX- and CFA-PTX-immunized mice compared to naïve animals, and only these are listed. [file 2045-8118-9-15-S7.pdf]

Additional file 7

Gene	MOG-CFA/PTX CP Epithelium D15<br><br>Avg % expn. ±sem	CFA/PTX CP Epithelium D15<br><br>Avg % expn. ±sem	*p* value
**Bcl2**	5.33 ± 2.84	1.44 ± 0.35	0.165387
**Ccl2**	2.49 ± 1.5	0.043 ± 0.004	0.111899
**Ccr2**	14.64 ± 9.32	0.75 ± 0.19	0.159383
**Ccl5**	14.34 ± 6.76	0.84 ± 0.27	0.062775
**Cd34**	8.63 ± 4.01	1.12 ± 0.36	0.076404
**Cd86**	3.34 ± 1.63	0.18 ± 0.02	0.069187
**Csf1**	11.07 ± 6.81	0.60 ± 0.11	0.125791
**Ece1**	2.40 ± 0.64	0.92 ± 0.33	0.056739
**Fas**	0.55 ± 0.32	0.09 ± 0.01	0.147418
**Hmox1**	4.05 ± 2.12	1.01 ±0.16	0.149136
**Hprt1**	302.55 ± 180.18	25.81 ±3.99	0.125903
**Il15**	14.86 ± 7.45	1.29 ±0.26	0.081535
**Il18**	3.38 ± 1.35	1.26 ± 0.34	0.128452
**Il1b**	1.78 ± 1.17	0.08 ±0.03	0.143367
**Nfkb1**	50.42 ± 24.19	4.26 ± 1.36	0.07184
**Stat4**	0.85 ± 0.51	0.06 ±0.02	0.121514
**Tfrc**	40.39 ± 21.63	1.12 ± 0.39	0.082216
**Vcam1**	15.41 ± 6.32	3.52 ±0.49	0.075254
**Vegfa**	9.69 ± 5.56	1.24 ± 0.4	0.129943
