# Supplementary material for: Active induction of experimental autoimmune encephalomyelitis by MOG35-55 peptide immunization is associated with differential responses in separate compartments of the choroid plexus
Source: Fluids Barriers CNS. 2012 Aug 7;9:15. doi: 10.1186/2045-8118-9-15 (PMC3493354; doi:10.1186/2045-8118-9-15)
Supplement: Additional file 8 — Genes similarly up-regulated in CP epithelium from both MOG-CFA/PTX- and CFA-PTX-immunized mice at day 15 p.i. Relative mRNA expression values of 93 immune-related genes were determined by immuno-LCM/TLDA in CP epithelium from immunized and naïve mice at day 15 p.i. At this later time-point, 8 immunization-induced genes were similarly stimulated in both MOG-CFA/PTX- and CFA-PTX-immunized mice compared to naïve animals, and only these are listed. [file 2045-8118-9-15-S8.pdf]

Additional file 8

Genes modulated similarly in CP Epithelium of MOG-CFA/PTX and CFA/PTX at day 15 p.i.
Gene name
Ccl19
Ccr7
Cd4
Cd8a
Cxcr3
Fn1
Sele
Selp
